# Novel Secondary Ion Mass Spectrometry Methods for the Examination of Metabolic Effects at the Cellular and Subcellular Levels

**DOI:** 10.3389/fnbeh.2020.00124

**Published:** 2020-07-20

**Authors:** Elisa A. Bonnin, Silvio O. Rizzoli

**Affiliations:** ^1^Department of Neuro- and Sensory Physiology, Excellence Cluster Multiscale Bioimaging, University Medical Center Göttingen, Göttingen, Germany; ^2^Center for Biostructural Imaging of Neurodegeneration (BIN), University Medical Center Göttingen, Göttingen, Germany

**Keywords:** cellular behavior, SIMS, isotope labeling, high-resolution imaging, nanoscale

## Abstract

The behavior of an animal has substantial effects on its metabolism. Such effects, including changes in the lipid composition of different organs, or changes in the turnover of the proteins, have typically been observed using liquid mass spectrometry methods, averaging the effect of animal behavior across tissue samples containing multiple cells. These methods have provided the scientific community with valuable information, but have limited resolution, making it difficult if not impossible to examine metabolic effects at the cellular and subcellular levels. Recent advances in the field of secondary ion mass spectrometry (SIMS) have made it possible to examine the metabolic effects of animal behavior with high resolution at the nanoscale, enabling the analysis of the metabolic effects of behavior on individual cells. In this review we summarize and present these emerging methods, beginning with an overview of the SIMS technique. We then discuss the specific application of nanoscale SIMS (NanoSIMS) to examine cell behavior. This often requires the use of isotope labeling to highlight specific sections of the cell for analysis, an approach that is presented at length in this review article. We also present SIMS applications concerning animal and cell behavior, from development and aging to changes in the cellular activity programs. We conclude that the emerging group of SIMS technologies represents an exciting set of tools for the study of animal behavior and of its effects on internal metabolism at the smallest possible scales.

## Introduction

Recent years have produced large advances in the analysis of the general aspects of proteins, including “omics” studies for protein abundance (Antonelli et al., 2019; Noor et al., 2019), analysis techniques for the abundance of mRNA (Washburn et al., 2019), analyses of protein translation rates (Riba et al., 2019; Sharma et al., 2019) and much more. Similarly, substantial progress has also been made in microscopy techniques for imaging the location of proteins and the general morphology of cells. For example, optical microscopy techniques have been able to observe cellular processes at the nanoscale (Schermelleh et al., 2019). X-ray imaging and tomography techniques are also progressing and are useful tools in the cellular study (Weber et al., 2019; Zhang, 2019). These encouraging trends suggest that the organization and spatial configuration of cells will be examined efficiently in the next few years. Live fluorescence imaging complements these approaches by providing information in the temporal domain, albeit only in the short term, from seconds to hours. Overall, despite the power of these tools, the long-term temporal domain remains relatively unclear, since none of these technologies provide sufficient information on the cellular activity over days or longer.

The examination of cellular activity at various time scales is critical for the study of much of animal behavior, as it is well-established that the metabolic processes of a cell influence behavior and vice versa (for example Kohsaka et al., 2007; Leulier et al., 2017). This has been recognized decades ago, and several technologies have therefore been introduced. They typically involve measuring the composition of cells suspended in solution, using conventional biochemical tools or in-solution mass spectrometry approaches. Unlike many of the modern techniques mentioned above, these tools provide information on processes and rates averaged across multiple cells. These methods are therefore limited in resolution and are unable to fully describe metabolic processes at the cellular and subcellular scales.

A modern solution to this problem comes from new advances in mass spectrometry imaging techniques, particularly in the field of secondary ion mass spectrometry (SIMS). SIMS analysis, which makes use of instruments such as time-of-flight secondary ion mass spectrometry (ToF-SIMS) and nanoscale secondary ion mass spectrometry (NanoSIMS), allows for the imaging of individual cells, with resolutions up to the 100 s of nanometers. When coupled with isotope labeling and other correlative microscopy techniques, SIMS analysis represents an exciting new avenue for the examination of animal behavior at the cellular and subcellular scales, across the long-term temporal domain.

In this review article, we present an overview of the SIMS technique and its application for the examination of the behavior of individual cells. We begin with a general overview of SIMS methodology and later focus on the specific application of SIMS in cellular studies. We present three case studies that have used NanoSIMS to examine the behavior of individual cells, and we also discuss the broader applications of SIMS in biological and environmental studies. These topics aim to inform the reader about the advantages and limitations of these novel techniques, which will likely continue to expand in the future, as creative applications for SIMS technology are continually developed.

## SIMS Analysis

**Figure 1:**
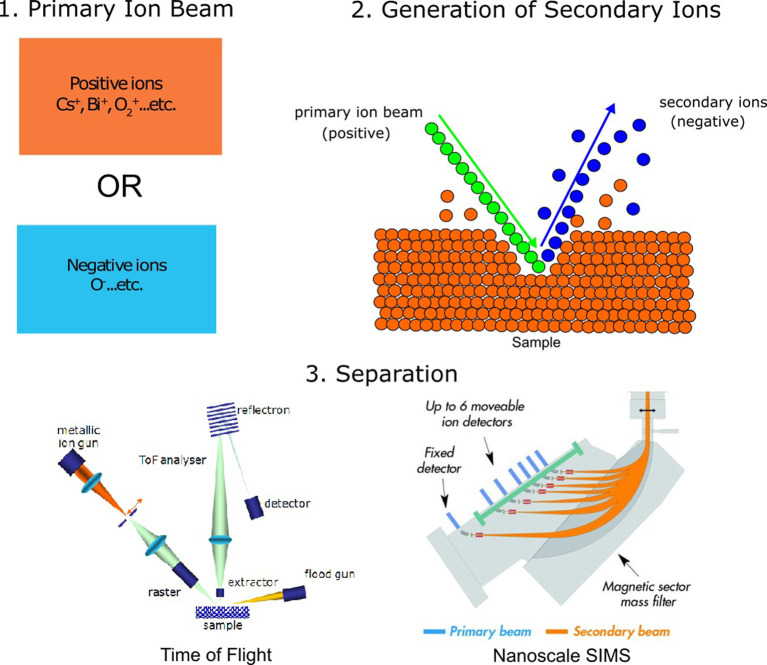
General principles of secondary ion mass spectrometry (SIMS) analysis. (1) SIMS analysis begins by making use of a primary ion beam, which can either be made of positive ions or negative ions. (2) The primary ion beam strikes the analysis surface during sputtering, producing secondary ions. (3) Secondary ions are accelerated towards the detector, either through a flight tube [time-of-flight SIMS, ToF-SIMS; image adapted with permission from Mazel and Richardin (2009) or through magnetic separation using a quadrupole (nanoscale SIMS, NanoSIMS), image adapted with permission from Nuñez et al. (2018)].

SIMS refers to measurement techniques wherein a selection of samples under a high vacuum is removed using a beam consisting of primary ions. This process is known as “sputtering.” Sputtering using the primary ion beam generates secondary ions, ionized atoms, and molecules which are then ejected from the analysis chamber and conveyed into a mass spectrometer for analysis (Figure 1; Fearn, 2015; Nuñez et al., 2018). While all SIMS techniques share these initial similarities, they differ slightly in the source of the primary ions, the voltage with which the ion beam strikes the sample, the amount of sample removed, and the separation of ions for detection. Two of the most common types of SIMS instruments used for imaging are time-of-flight SIMS (ToF-SIMS) and nanoscale SIMS (NanoSIMS). Matrix-assisted laser desorption/ionization combined with mass spectrometry (MALDI-MS), while not strictly a SIMS technique, is also often used to image biological samples and is mentioned here for comparison. For more information on MALDI and other mass spectrometry imaging methods, we refer the reader to the following reviews and studies: Hanrieder et al. (2013); Passarelli and Ewing (2013); Petras et al. (2017); Buchberger et al. (2018); and Xiao et al. (2020).

SIMS analyses are generally used to create images, where each pixel of the image contains a mass spectrum with chemical information of the analyte of interest. As such, the spatial resolution of this technique is typically correlated to beam diameter and pixel density. Previous studies have discussed methods of estimating the spatial resolution of SIMS by imaging over a known edge (Senoner et al., 2004). In contrast with the spatial resolution, the mass resolution of SIMS refers to the ability of the instrument to distinguish between adjacent masses. This is also referred to as the mass resolving power, or M/ΔM, where M is the mass being measured and ΔM is the mass difference resolvable between two peaks of interest (Hillion et al., 1993). A summary of expected sensitivities, mass resolutions, and spatial resolutions of SIMS techniques can be found in Table 1. The major differences between the three mentioned imaging techniques have to do with their mode of detection and the choice of the primary ion source, which affects both sensitivity and spatial resolution.

**Table 1:** Comparison of secondary ion mass spectrometry (SIMS) techniques with other mass spectrometry imaging techniques.

Instrument	Primary Ion Source	Spatial Resolution	Mass Resolution (M/ΔM)	Upper Mass Limit
NanoSIMS	Ion beam (Cs^+^ or O^−^)	<50 nm (Cs^+^), <200 nm (O^−^)	10,000^1^	Atomic and diatomic (e.g: CN^−^) ions^1^
ToF-SIMS	Ion beam (Bi_3^+_, C_60^+_, Ar_n^+_…etc.)	200 nm–5 μm (dependent on source)	>10,000^2^	1,500–2,500 Da^3^
MALDI-MS/MS	Laser light	1–150 μm^4^ (typically 10 μm)	>1,000,000^5^ (using Fourier Transform ion cyclotron resonance mass spectrometry)	20 kDa^6^

*^1^Hillion et al. (1993); Nuñez et al. (2018); ^2^Boxer et al. (2009); ^3^Fearn (2015) ^4^Hanrieder et al. (2013); ^5^Dilillo et al. (2017); ^6^Jones et al. (2014)*.

Unlike many other instrumental methods, which require the use of individual labels such as antibodies to mark specific proteins for analysis, SIMS images can be acquired without the use of labels (Hanrieder et al., 2013; Passarelli and Ewing, 2013). This is in contrast to techniques such as fluorescent noncanonical amino acid tagging (FUNCAT), which always requires the use of special labels (Dieck et al., 2012). In FUNCAT experiments a cellular metabolite, such as the amino acid methionine, is replaced with a noncanonical one, which is incorporated into proteins through the metabolic processes of the cells (i.e., protein synthesis). The typical noncanonical amino acids used in these procedures bear either an azide or an alkyne and are later revealed by a specific reaction to a modified fluorophore, termed a click reaction (Dieterich et al., 2010). This procedure is readily applied to cell cultures, where amino acids in the cell medium can be replaced easily with noncanonical ones but it is difficult to perform with animals. The ability of SIMS analyses to obtain information on the location of different structures without the use of these tools is a notable advantage of this method, although there are instances in which these labels can be used to enhance SIMS analysis (for example see Kabatas et al., 2015, 2019a,b; Vreja et al., 2015). These methods will be discussed in more detail later in this review article.

Within SIMS techniques, a difference exists between “static” and “dynamic” SIMS, where “static” SIMS refers to SIMS analyses that use a low primary ion dose (generally less than 10^13^ ions cm^−2^) which removes only 1% of atomic sites from the sample surface (Benninghoven, 1969). In contrast, “dynamic” SIMS refers to SIMS techniques that remove relatively large amounts of material. “Static” SIMS techniques are useful for obtaining molecular information, as the chemical bond between molecular fragments is more likely to be preserved at low primary ion doses, while “dynamic” SIMS techniques do not preserve chemical bonds, and are useful for providing high spatial resolution data on the distribution of elements in a sample (Cannon et al., 2000).

As the name implies, time-of-flight secondary ion mass spectrometry (ToF-SIMS) uses time-of-flight as a detection mode, where ions are accelerated through a flight chamber and are separated based on the time it takes for ions to reach the detector. Using ToF-SIMS, spatial resolutions up to 200 nm are possible (Fearn, 2015). In a typical dual-beam ToF-SIMS instrument, a primary ion gun is used to generate secondary ions for analysis, while a second “sputter” ion gun is used for depth profiling. There are a variety of primary ion sources available for ToF-SIMS, including the bismuth (Bi_n_^+^; Nygren et al., 2005; Touboul et al., 2005b), gold (Au^++^, Au^+^, Au_2_^+^; Walker and Winograd, 2003) and gallium (Ga; Vickerman, 2001) sources, the buckminsterfullerene (C_60_^+^) source (Wong et al., 2003), and the argon gas cluster (Ar_n_^+^) source (Rabbani et al., 2011). Sputtering with the primary ion source yields charged secondary ions, which are then accelerated into the flight chamber.

ToF-SIMS can be operated in static mode, where <1% of material is removed. This is often ideal for biological samples, however, to maintain static mode, higher concentrations of the analyte of interest are required to maintain spatial resolution. Besides, ToF-SIMS can also be operated under different conditions and configurations, by changing the source of the primary ion beam. For example, the C_60_^+^ source, the Ar_n_^+^ source, and other polyatomic ion sources have recently been used to generate 3D chemical images, which can provide more detailed information from multi-layered samples (for example see Brison et al., 2013; Fletcher, 2015). This relatively new capability has expanded the utility of ToF-SIMS in examining biological samples.

ToF-SIMS is a flexible technique that, depending on the current of the primary ion beam, can obtain information from molecular fragments or atomic information. Thus, ToF-SIMS is a useful tool for mapping the distribution of elements, ions, and molecules in unlabeled samples. In contrast, NanoSIMS cannot be operated in static mode. Instead, NanoSIMS is always operated in dynamic mode. While ToF-SIMS can generate both atomic and molecular fragments as secondary ions, NanoSIMS only generates secondary ions at the atomic (and occasionally diatomic) scale. In the absence of molecular secondary ions, stable isotope measurements in NanoSIMS can be used to infer molecular distribution (for example see Pett-Ridge and Weber, 2012).

The NanoSIMS method is typically used with one of two sources, the cesium source (Cs^+^), which enhances the ionization of negative secondary ions, and the oxygen source (O^−^ or $O_{\text{2}}^{-}$), which enhances the ionization of positive secondary ions. A new radio frequency (RF) oxygen source has been developed, which has increased the sensitivity and long-term stability of this mode (Malherbe et al., 2016). The Cs^+^ source, which has a maximum spatial resolution of <50 nm, is used to examine elements that more readily ionize into negative secondary ions, such as H, C, N, O, F, P, S, and Cl. The oxygen source, in contrast, is used to examine elements that more readily ionize into positive secondary ions, such as Li, Na, Mg, Ca, and some transition metals (Nuñez et al., 2018).

The NanoSIMS makes use of both an electrostatic and magnetic sector. Ions are deflected into six movable detectors and one stationary detector, which allows for a total of seven species to be detected during each analysis. The need to decide on a maximum of seven species at a single time is one limitation of the NanoSIMS instrument. Also, negative and positive ions cannot be collected within the same analysis, necessitating that researchers choose analytes that can be measured with either the Cs^+^ source or the O^−^ source respectively. Also, NanoSIMS cannot be operated in static mode, and as such, provides information only on the distribution of elements and small molecular fragments such as CN^−^. Despite these limitations, the high spatial resolution and mass resolution of NanoSIMS and its ability to collect reliable isotopic information make it a highly useful technique.

Both ToF-SIMS and NanoSIMS rely heavily on appropriate sample preparation to ensure the quality of these analyses. In particular, SIMS analysis requires the preparation of thin, flat surfaces which are then placed on the conductive material. The methods for generating these samples vary depending on the type of sample and the nature of the study. For example, cryo-sectioning with a microtome and directly applying the sections to a conductive surface is common for tissue analysis (Amstalden van Hove et al., 2010), whereas other SIMS samples are embedded in various heat and/or vacuum resistant epoxies before sectioning and placing on silicon wafers (Saka et al., 2014). In cases where conductivity is difficult to achieve, such as with geological samples, coating with a thin layer of a conductive material such as gold is also common (Bonnin et al., 2019). A full discussion of sample preparation is beyond the scope of this review, however, we refer the reader to the following reviews and studies, which cover sample preparation methods in more detail: Goodwin (2012); Hanrieder et al. (2013); Passarelli and Ewing (2013); Wang et al. (2014); Fearn (2015); Dong et al. (2016); Nuñez et al. (2018); and Buchberger et al. (2018).

MALDI, an instrumental technique that ionizes the sample using a laser energy absorbing matrix, is typically used to analyze large molecular species (up to 10,000 Da) which cannot be imaged using NanoSIMS. However, MALDI has an imaging resolution in the micrometer range, which is substantially poorer than either of the SIMS techniques (Schwamborn and Caprioli, 2010; Passarelli and Ewing, 2013; Buchberger et al., 2018). While the spatial resolution of this technique has been improving (Hanrieder et al., 2013), and this technique is useful for mapping proteins and peptides, its low spatial resolution makes it less suitable for the subcellular and cellular scales focused on in this review, and so we primarily discuss applications of ToF-SIMS and NanoSIMS.

## Analyzing Unlabeled Samples with SIMS

Both NanoSIMS and ToF-SIMS instruments can be used to examine samples that are prepared for analysis and introduced into the SIMS without any elemental or isotopic labeling. To successfully use SIMS to image these samples, the analyte of interest must be sufficiently distinct from the surrounding matrix. For example, the NanoSIMS technique has been used to examine the distribution of selenium in cereal grain and arsenic in rice (Moore et al., 2010). Because arsenic and selenium are not major components of the structure of cereals and rice, SIMS can be used to detect areas where these elements can be found in high concentrations. The NanoSIMS has also been used to examine the origin of dust particles in Luxembourg (Krein et al., 2008) and to examine Ca and P in the axonal and glial regions of mice to study the response of the central nervous system to neurotrauma (Lozić et al., 2014). The latter study revealed that Ca microdomains not associated with P rapidly decrease after injury, while Ca microdomains associated with P are unaffected. In recent years, NanoSIMS has also become useful in the study of ancient life. Typically, in these studies, samples are examined for traces of organic material, using chemical features that indicate biological activity. For example, measuring C, N, and S in fossil samples taken from the Omdraaivlei Formation in South Africa (Kaźmierczak et al., 2016) allowed researchers to identify evidence of ancient organisms.

The majority of NanoSIMS techniques, however, take advantage of its high mass resolution by combining it with isotope labeling techniques, an approach that will be discussed in detail in the next section. For further reference on the use of NanoSIMS for analyzing biological samples, both labeled and unlabeled, see Nuñez et al. (2018). In contrast, ToF-SIMS is often used in examining unlabeled samples. For example, ToF-SIMS has been used to examine the incorporation of calcium into silicon-based bone grafts, an important precursor to bone regeneration (Wang et al., 2014). In conjunction with atom probe tomography (APT), ToF-SIMS has also been used to examine the organic-mineral interface in the calcium carbonate shells of foraminifera, a type of marine zooplankton, which has implications for studies of biomineralization (Branson et al., 2016; Bonnin et al., 2019). In a similar vein, ToF-SIMS analyses have also been conducted on tissues and lipids to examine lipid biomarkers of diseases (Touboul et al., 2005a; Debois et al., 2009; Kezutyte et al., 2013) and to examine the distribution of cholesterol in brain tissue, which is relevant for studies of Alzheimer’s disease (Lazar et al., 2013).

While this review is primarily focused on the use of isotope labeling to examine subcellular metabolic effects, we note that valuable insights into cell behavior can still be gained through the use of unlabeled samples. For example, a study by Philipsen et al. (2018) used ToF-SIMS to examine major lipids in the brains of specimens of the fruit fly *Drosophila melanogaster* that had been exposed to cocaine and methylphenidate (MPH), a common ADHD medication. In this experiment, three- to four-day-old male flies were transferred to yeast paste containing either 15 mM cocaine or 50 mM MPH for 3 days. The fly heads were then detached and brain samples analyzed using ToF-SIMS.

**Figure 2:**
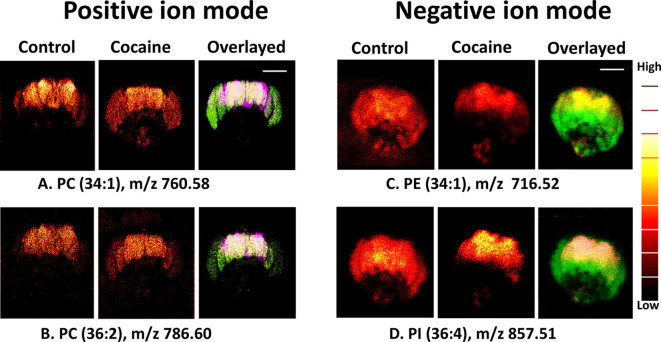
Distribution of biomolecules in the *Drosophila* brain before and after cocaine treatment by ToF-SIMS in positive and negative ion modes. Image area: 800 × 800 μm^2^ and 256 × 256 pixels; pixel size, ~3 μm. Overlaid images: **(A,B)** purple, control brain; green, cocaine-treated brain (positive ion mode); **(C,D)** green, control; red, treated brain (negative ion mode). Scale bar: 200 μm. A color “thermal” scale is shown. This shows marked differences in the distribution of lipids after the introduction of cocaine, particularly in the negative ion mode. This may indicate that behavioral changes induced by cocaine can be related to changes in lipid distribution, and can be observed through SIMS. Reprinted with permission from Philipsen et al. (2018).

*Philipsen et al*. found that lipid distribution changed after the administration of cocaine, with lipids that form positive ions becoming visibly more abundant in the central brain and optical lobes after cocaine administration and lipids that form negative ions becoming less abundant (Figure 2). This suggests that cocaine use may change the chemical structure of the brain. While MPH also altered the lipid distribution of the *Drosophila* brain, the alterations caused by MPH were strikingly opposite to those caused by cocaine. Because cocaine and MPH also have opposite behavioral effects, with MPH enhancing cognition, memory, and behavior while cocaine decreases attention, learning, and memory, the results of this study imply a link between brain lipid distribution and cognition.

Similarly, a recent study by Dowlatshahi Pour et al. (2019) used ToF-SIMS on unlabeled brain samples to measure hippocampal zinc, which is an essential trace element in many proteins, but which also acts as a neurotoxin in high concentrations. By using ToF-SIMS to examine zinc-related compounds, an act which necessitates using static SIMS to preserve chemical bonds, the researchers were able to observe that zinc compounds in the rat hippocampus increase in concentration after acute brain injury. Because these compounds are all from bound zinc species, their findings challenge the idea that the accumulation of free zinc in synaptic vesicles is the main source of neuronal degeneration after traumatic brain injuries. These studies highlight the fact that valuable insights into cell behavior can be obtained from unlabeled samples, particularly when using ToF-SIMS.

## Combining SIMS Analysis with Isotope Labeling

Isotope labeling is a powerful tool that can be used to highlight areas of interest with high resolution and precision. In general, isotope labeling is used in SIMS analysis in one of two ways, either to highlight a specific area of interest or to mark a particular experimental time. In both cases, rare stable isotopes are used because the rare isotope is easily distinguished from the surrounding matrix, and the ratio of rare isotope to common isotope in nature is well-constrained. Because NanoSIMS can differentiate between stable isotopes of the same element with high resolution, using SIMS analysis on an isotopically labeled sample allows the researcher to see the labeled area clearly in the resulting intensity map (Steinhauser et al., 2012).

**Figure 3:**
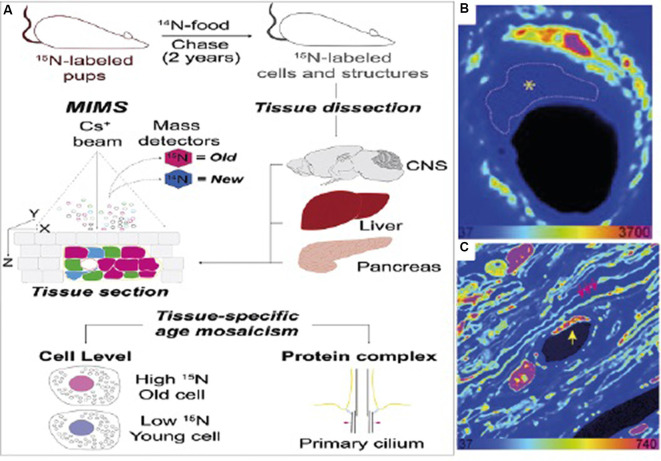
**(A)** Sample measurement scheme for an isotope labeling NanoSIMS experiment. In this case, the isotope label used is ^15^N, which is used to mark “older” cells. **(B)** NanoSIMS image from the optic nerve head (ONH) of the labeled mouse. Here, bright colors refer to older, labeled cells. The dashed line refers to an endothelial cell present in the electron microscopy image which does not have any discernible features in the NanoSIMS image. **(C)** NanoSIMS image of two capillaries in the ONH of a 6-month chase mouse. An endothelial cell nucleus (yellow arrow) and myelin sheaths (pink arrows) are indicated. A pericyte nucleus is visible to the left of the capillary lumen. Labeled cells are visible in comparison to unlabelled cells, illustrating the usefulness of isotope labeling methods applied to SIMS. Reprinted from Arrojo e Drigo et al. (2019) with permission fromElsevier.

Isotope labeling is particularly useful in biological studies because rare isotopes are processed by organisms in the same way as common isotopes are, and are incorporated into the organism’s biomass in the same way as ordinary organic material. This makes it possible to label tissues and other parts of the organism by introducing the stable isotope label into the organism’s food (Steinhauser and Lechene, 2013). If the amount of time it takes an isotope label to be incorporated into the tissue is known, an isotope label or several isotope labels can be used to mark time points throughout an experiment. For example, a researcher can introduce the isotopic food at the start of an experiment, so that only cells produced during the experiment are labeled (for example see Hassouna et al., 2016), or a researcher can provide isotopic food for a certain amount of time as part of a pulse-chase experiment. Figure 3 shows one example of such an isotope labeling scheme, taken from Arrojo e Drigo et al. (2019). In this experiment, described in more detail later, ^15^N food is used at the beginning of the organism’s life to mark old material. The label can be seen very clearly in the subsequent NanoSIMS images.

Isotope labeling has also been used in the fields of oceanography and paleoclimatology, to label portions of the skeletons of corals and other calcifying organisms. Many of these organisms will be affected by ocean acidification and climate change in the future, and some of these organisms are also useful for paleoclimate studies because the chemical composition of their shells changes in measurable ways as the surrounding environment changes. For both these applications, it is beneficial to understand how the organism’s skeleton or shell is formed. Isotope labeling provides valuable insights into this process. For example, Brahmi et al. (2012) demonstrated how labeling a coral skeleton with ^86^Sr can be used to calculate average extension rates, allowing researchers to examine the rate of biomineralization in these organisms.

Furthermore, NanoSIMS has seen increasing use in the field of environmental microbiology, where the instrument’s ability to track the flow of biologically active elements such as nitrogen, carbon, and iron is greatly useful. By using NanoSIMS and isotope labeling, the distribution of these cellular activities (for example, carbon and nitrogen fixation) can be examined (Behrens et al., 2012). Carbon and nitrogen fixation in algae from marine and freshwater habitats, for example, can be examined using ^13^C and ^15^N labels to examine how carbon and nitrogen are fixed through ecosystems. The use of SIMS to measure environmental processes is further detailed in the review by Behrens et al. (2012).

The versatility of isotope labeling means that it can be used to examine questions on the scale of whole organs or organisms, but SIMS and isotope labeling can also be used at the subcellular level to examine processes within cells. For example, NanoSIMS has been used to track the uptake of isotopically labeled human proteins by microbes in the human intestine (Berry et al., 2013), to measure the anabolic activity of *Staphylococcus aureus* by tracking deuterium (^2^H) and ^15^N (Kopf et al., 2016), and to examine dividing cells in the small intestine (Steinhauser and Lechene, 2013).

While isotope labeling is a common part of SIMS experiments involving microbes, rats, or mice, there is growing evidence that suggests that isotope labeling and SIMS may also be used to examine human metabolism and cell behavior, by the administration of ^15^N thymidine to a human volunteer (Steinhauser and Lechene, 2013). This is an exciting avenue for research, and will likely become more prominent in future years if it can be proven that the patient suffers no ill effects from the administration of these isotopically enriched chemicals. In further sections of this article, we present three examples of the combination of isotope labeling and NanoSIMS to examine the behavior of cells in various organs.

## Sample Case 1: Brain

Hassouna et al. (2016) used a combination of NanoSIMS and ^15^N labeling during an investigation on the effects of recombinant human erythropoietin (EPO) on cognitive performance in mice. The goal of this study was to examine whether EPO, which has been suggested to improve cognitive performance in cases such as schizophrenia, multiple sclerosis, major depression, and bipolar disease, is linked to neurogenesis. To investigate this, the authors first injected male mice intraperitoneally with either recombinant human EPO or a placebo, beginning at the age of 28 days. Isotope labeling was achieved by feeding the mice food pellets labeled with ^15^N, also beginning at the age of 28 days. Ensuring that the ^15^N labeled food was coincident with the start of the experiment is important, as it ensured that only cells produced during the experimental period were labeled with ^15^N. In this case, ^15^N serves as a marker for experimental cells.

The mice included in the NanoSIMS study were given either EPO or placebo injections every other day over 3 weeks. After three weeks of feeding, EPO and ^15^N-leucine feeding stopped simultaneously. One week later, mice were anesthetized and brains removed. Brain slices were embedded in resin and imaged using a combination of fluorescence microscopy and NanoSIMS. Other mice were separated into groups for cognitive experiments.

**Figure 4:**
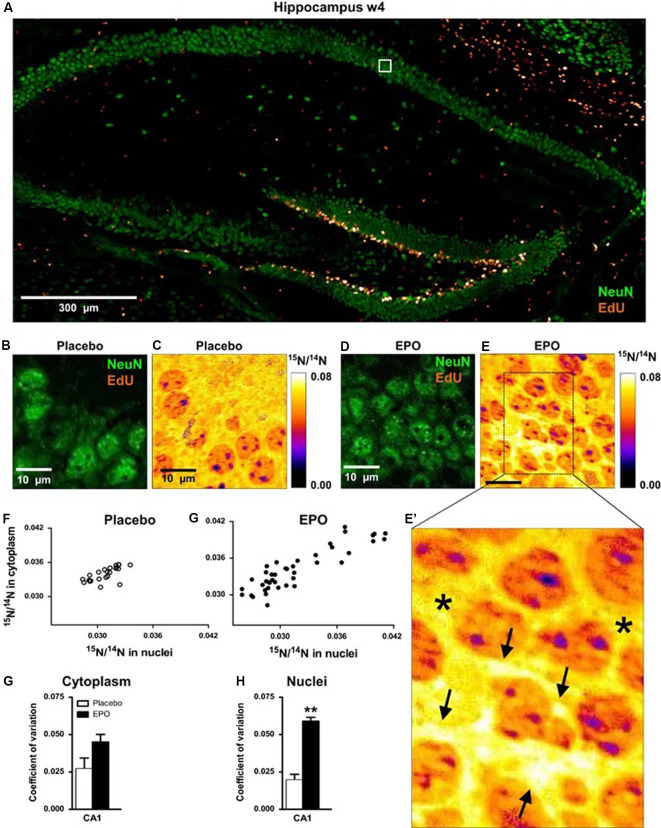
^15^N-leucine incorporation in CA1 pyramidal neurons evaluated by NanoSIMS. **(A)** Overview of the hippocampus showing dense proliferation signals (EdU) in the dentate gyrus. The white square illustrates the EdU signal-free area in the pyramidal layer, analyzed by NanoSIMS. **(B,C)** Illustration of samples following placebo treatment; **(D,E,E’)** Illustration of samples following erythropoietin (EPO) treatment; arrows in panel **(E’)** point to newly generated neuronal cell mass with high^15^N/^14^N ratio; stars in **(E’)** denote “control” signal in cytoplasmic regions of older neurons. **(F,G)** Scatter plots of ^15^N/^14^N ratios in pyramidal neurons in both treatment groups. **(H,I)** Coefficient of variation of ^15^N/^14^N ratios in cytoplasm and nuclei of pyramidal neurons (*n* = 3 for both groups). All bar graphs are shown as mean ± s.e.m.; **P < 0.01 (unpaired two-tailed *t*-test). ^15^N incorporation appears higher in EPO-treated mice, indicating increased protein synthesis within pyramidal neurons treated with EPO. These observations thus show that EPO may contribute to central nervous system regeneration in adults, which may have behavioral and cognitive implications. Reproduced with permission from Hassouna et al. (2016).

In this study, the authors used NanoSIMS was used to identify pyramidal neurons with higher than average ^15^N incorporation (Figure 4). Because higher than average ^15^N could only come from the ^15^N leucine feeding, cells with higher than average ^15^N can be assumed to correspond to areas of induced protein synthesis. By comparing the brains of the EPO-treated mice and the placebo-treated mice, the researchers found that ^15^N incorporation was higher in EPO-treated mice, suggesting that treatment with EPO resulted in increased protein synthesis within pyramidal neurons. This was supported by stereology (cresyl violet) and CTIP2 staining, both of which showed an increase in cell number in EPO-treated brains. When combined with cognitive studies of EPO treated mice, the researchers were able to conclude that EPO can not only improve cognition in mice and humans but also result in increased amounts of neurogenesis from inconspicuous local precursors. This is relevant for studies of central nervous system regeneration in adults, as EPO expression has been observed to be linked to brain injury, and presumably to recovery from injury.

## Sample Case 2: Heart

NanoSIMS and pulse-chase experiments using isotope labeling have also been used to obtain valuable information about the renewal of mammalian heart cells, as described in Senyo et al. (2013) which examined the differentiation of progenitor cells to cardiomyocytes. This study sought to answer a fundamental question about the formation of cardiomyocytes in adult mammals—specifically, whether stem cell activity could result in a high rate of renewal of cardiomyocytes, or whether new cardiomyocytes are born at a low rate, derived from the division of pre-existing cardiomyocytes.

To answer this question, the authors first administered ^15^N-labeled thymidine for 8 weeks to three age groups of mice—newborn (4 days), young adult (10 weeks), and old adult (22 months). The authors chose these periods to examine the formation of cardiomyocytes at different ages. Because, newly formed cells would be labeled with ^15^N, the authors could analyze samples using NanoSIMS and examine which cells showed higher than average ^15^N labeling, similar to the method used in Hassouna et al. (2016).

**Figure 5:**
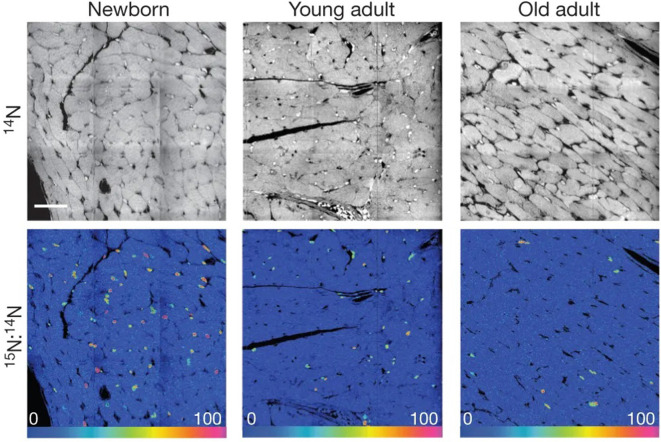
[^15^N]Thymidine was administered for 8 weeks to mice of different ages: newborn, starting at postnatal day 4; young adult, starting at 2 months; old adult, starting at 22 months. Top, ^14^N mass images show histological details. Bottom, ^15^N:^14^N hue–saturation–intensity images show ^15^N^+^ nuclei. Mosaics are constructed from nine tiles, 60  μm each. Scale bar: 30 μm. These data indicate a decrease in ^15^N-labeled cells with age, which demonstrates that DNA synthesis in cardiomyocytes decreases with age under normal conditions. In this case, the ^15^N label acts to mark DNA synthesis occurring after the administration of the isotope label. Reprinted by permission from Senyo et al. (2013).

Following NanoSIMS analyses on these samples, the authors found that in the newborn group, more than half of the cardiomyocytes showed ^15^N labeling. This suggested a large concentration of newly formed cells, consistent with the observation that cardiomyocytes continue to synthesize DNA and develop after birth. However, in the young adult group, the frequency of ^15^N-labeled cardiomyocytes decreased by 66-fold. In the old adult group, the frequency of the ^15^N label in cardiomyocytes further decreased. This indicates a decrease in DNA synthesis in cardiomyocytes with age under normal conditions (Figure 5). Further examination with ^15^N thymidine labeling on double-transgenic MerCreMer/ZEG mice showed that during aging, most new cardiomyocytes are derived from existing cardiomyocytes.

SIMS was also used to examine the effect of myocardial injury on cardiomyocyte renewal. For this experiment, a group of mice underwent surgery, during which they were subjected to myocardial infarction. A second, control group of mice also underwent surgery without myocardial infarction. Both groups were continuously labeled with ^15^N thymidine for over 8 weeks. In the mice that had sustained an injury, the frequency of labeled cardiomyocytes increased significantly in the area surrounding the infarction site, something that was not observed in the control group. This indicates that in those 8 weeks, cardiomyocyte division increased in the injured mice. This suggests that cardiomyocytes do have some ability to divide and re-enter the cell cycle, particularly after injury, however, the results of the overall study suggest that the majority of DNA synthesis in cardiomyocytes still occurs in pre-existing cells. By using a combination of SIMS and isotope labeling, the authors were able to conclude that cardiac progenitor cells do not significantly contribute to the formation of cardiomyocytes in mammals.

## Sample Case 3: Inner Organs

The combination of isotope labeling and SIMS analysis has also been applied to the question of cell age, as illustrated by Arrojo e Drigo et al. (2019). In this study, female mice were fed ^15^N labeled food before the introduction of a male. Feeding with the labeled food continued during pregnancy, including during mating, gestation, and lactation. Pups were then weaned onto ^14^N unlabeled food after either 21 or 45 days. Feeding with ^14^N food continued for a period of up to two years. This method results in cells that were produced during early development maintaining a ^15^N label, particularly if these cells did not divide or exchange material with ^14^N-rich cells. By using this method, the authors hoped to use SIMS to find especially long-lived cells that had maintained the ^15^N label from early development.

The authors reported age mosaicism in certain inner organs, including the central nervous system, pancreas, and liver, where some cells appeared long-lived, others new, and others of varying ages. In particular, the authors found that the liver and pancreas were composed of cells with different ages, particularly fibroblasts and endothelial cells. By having a subset of mice subjected to the experiment for six months, while another subset of mice was kept in experimental conditions for up to 18 months, the authors were also able to investigate the rate of turnover in various inner organs during that time.

**Figure 6:**
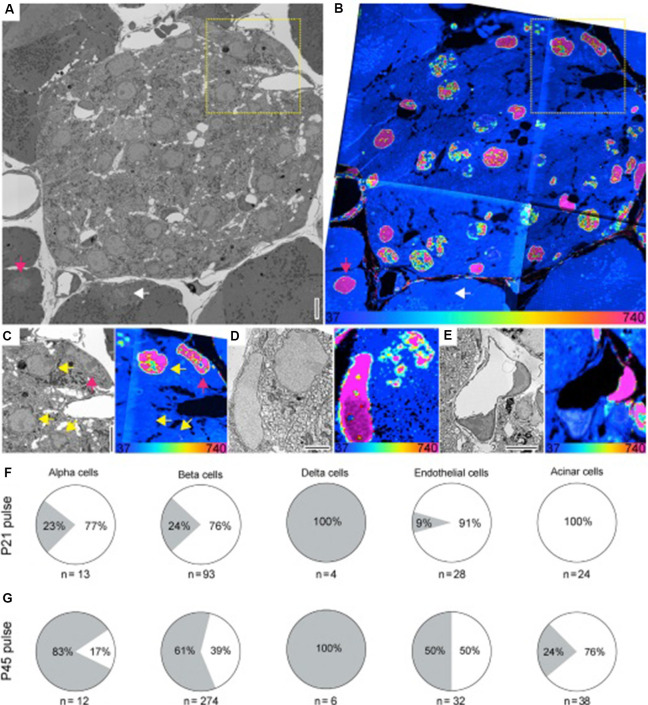
**(A,B)** SEM **(A)** and MIMS **(B)** of a cross-section of the islets of Langerhans (pancreas). Old and young acinar cells are indicated by the pink and white arrow, respectively. Yellow dotted box highlights cells shown in **(C)**. **(C)** Enlarged view of boxed region in **(A)** and **(B)**. SEM and MIMS of beta cells (yellow arrows) and an old alpha cell (pink arrow). **(D)** An old delta cell (left) next to a younger beta cell (top right). **(E)** SEM and MIMS of a young (bottom) and an old (top right) endothelial cell. Old pancreatic stellate cells are seen in the top and lower right corners. **(F)** Relative turnover in percentages of cells that are as old (gray) or younger (white) than L2 neurons from ^15^N-SILAM P21 mouse chased for 26 months. **(G)** Same as in **(F)**, but from a ^15^N-SILAM P45 mouse chased for 18 months. The total number of cells analyzed for each cell type is listed underneath each pie chart. At the bottom of the MIMS images, the heatmap shows the ^15^N/^14^N x 10^4^ and scaled with an HSI. Scale bars: 5 μm **(A,C,E)** and 2.5 μm **(D)**. In this case, the ^15^N label represents “older” cells. Thus, the ^15^N label can be used to identify long-lived cells in the pancreas, and using a pulse-chase experiment, shows the period during which cellular turnover is most likely to occur. Reprinted from Arrojo e Drigo et al. (2019) with permission from Elsevier.

In the liver, the authors reported that the majority of hepatocytes retained the ^15^N label during this period, remaining “older” cells. In contrast, stellate-like cells and sinusoid-like cells experienced significant turnover between 6 and 18 months of age, transitioning from mostly “old” cells to mostly “younger” cells. The pancreas, meanwhile, appears to be composed of cells of varying ages, with non-uniform turnover between alpha, beta, and delta cells. Comparing the cells from mice that had been weaned at 21 days with mice that had been weaned onto unlabeled food at 45 days, the authors found that a significant percentage of alpha and beta cells appear to be formed in the time between 45 and 21 days, while all delta cells appeared “old” in both conditions (Figure 6).

## Alternative Methods for Sample Labeling

Although SIMS techniques are useful recorders of chemical information, one enduring limitation of these techniques is that without an independent method for imaging the location of interest, it is difficult to determine exactly what cellular structure is being imaged. While certain cellular structures such as the nucleus are often obvious and easily distinguished in SIMS images, the same cannot be said for many organelles. One solution, then, is to combine SIMS imaging techniques with an optical microscopy technique. This technique, termed correlated optical and isotopic nanoscopy, or COIN, can allow researchers to obtain isotopic data from organelles and other structures which may not easily be recognized in SIMS (Saka et al., 2014). For detailed information on the application of COIN to biological samples, we refer the reader to Saka et al. (2014), which discusses the technique at length.

In brief, however, the proper application of COIN requires that the microscopy technique used is of sufficient resolution to correlate the organelle of interest with the isotopic signature. Thus, while confocal microscopy techniques are sufficient for organelles above the diffraction limit, smaller organelles and proteins would require super-resolution microscopy techniques for COIN to be usable. Also, COIN requires that a biological sample be prepared in a manner such that the same sample can be imaged by both techniques. This requires ensuring that the resin used is viable for both SIMS analysis and the chosen optical microscopy technique and that samples are cut to a thickness that optimizes resolution in both methods. It must also be possible to image the same location in both techniques, which would require a method of marking the area of interest without damaging the sample.

These requirements impose additional limitations on this technique. Other solutions have been proposed, such as the immunostaining of samples using antibodies coupled to isotopically pure metals (Angelo et al., 2014). In this manner, antibodies can be imaged through SIMS, which eliminates the need for correlative microscopy. However, antibodies coupled to metal tags tend to incorporate poorly into specimens. This undermines the high resolution of SIMS, resulting in a less precise image (Opazo et al., 2012; Ries et al., 2012).

To solve these issues, various methods using elemental and isotopic probes have been developed, which add enriched labels to proteins of interest. For example, labels enriched with ^19^F can be added to various proteins, which can then enable them to be analyzed both using NanoSIMS and using fluorescence imaging (Vreja et al., 2015). Similarly, areas of interest can be labeled by using boron-based probes, which then can be used to reveal the structure of proteins while other measurement channels can be used to examine other pertinent positive ions (Kabatas et al., 2019b). These methods allow for more flexibility when designing SIMS experiments, and alleviate some of the limitations of the SIMS techniques.

The use of correlative microscopy techniques and probes to better identify analysis regions in SIMS is an ongoing area of study and is a complex issue. The combination of SIMS, various probes and labels, and microscopy is not necessarily straightforward, and a full discussion of these tools would be more suited as the subject of its review. However, it appears clear that the development of new SIMS imaging techniques will remain correlated with advances both in microscopy and elemental probe techniques.

## Conclusions

The high resolution and sensitivity of SIMS make it a good tool for the analysis of behavior at the cellular and subcellular levels, particularly when coupled with isotope labeling techniques to highlight areas of interest. Advances in SIMS technology continue to make this a promising method for the analysis of cell structure, metabolism, and protein turnover, which can ultimately lead to changes in animal behavior. The various studies mentioned in this review showcase the potential of this technique for measuring behavioral and metabolic effects and also show other biological and environmental applications for these new and emerging techniques.

It should be noted, however, that the majority of these studies also made use of correlative microscopy techniques. Hassouna et al. (2016), for example, made use of fluorescence and staining, while Arrojo e Drigo et al. (2019) utilized electron microscopy to determine the location of cells. This highlights one enduring limitation of SIMS techniques, namely that without an independent method for imaging the location of interest, it is difficult to tell exactly what structure being analyzed. Because of this, the development of new SIMS techniques will likely remain correlated with advances in microscopy. However, as further developments continue in these areas, SIMS will likely remain a fundamental tool in cellular imaging well into the future.

## Author Contributions

EB and SR planned and wrote the manuscript.

## Conflict of Interest

The authors declare that the research was conducted in the absence of any commercial or financial relationships that could be construed as a potential conflict of interest.
